# Case reports: old-timers and evergreens

**DOI:** 10.1186/s13256-018-1889-3

**Published:** 2018-11-27

**Authors:** C. A. Koch, T. Fülöp

**Affiliations:** 1 Medicover GmbH, Berlin, Germany; 2 Carl von Ossietzky University of Oldenburg, Oldenburg, Germany; 3 Technical University of Dresden, Dresden, Germany; 4 University of Louisville, Louisville, KY USA; 5 Medical University of South Carolina, Charleston, USA

After more than 11 years of service to the *Journal of Medical Case Reports* (*JMCR*), I am writing these lines with mixed feelings, as this has been a wonderful journey and I am now becoming an “old-timer”… or evergreen (Fig. 1). First, I wish to thank the editor Prof. Michael Kidd for his leadership and trust in me as a deputy editor of the journal. Furthermore, I wish to thank all the deputy editors, especially Profs. Richard Rison and Jean Karl Soler for their collegiality. Together, and with the leadership of Prof. David Riley, we launched the CARE (CAse REport) guidelines in 2013 [1, 2].

**Fig. 1:**
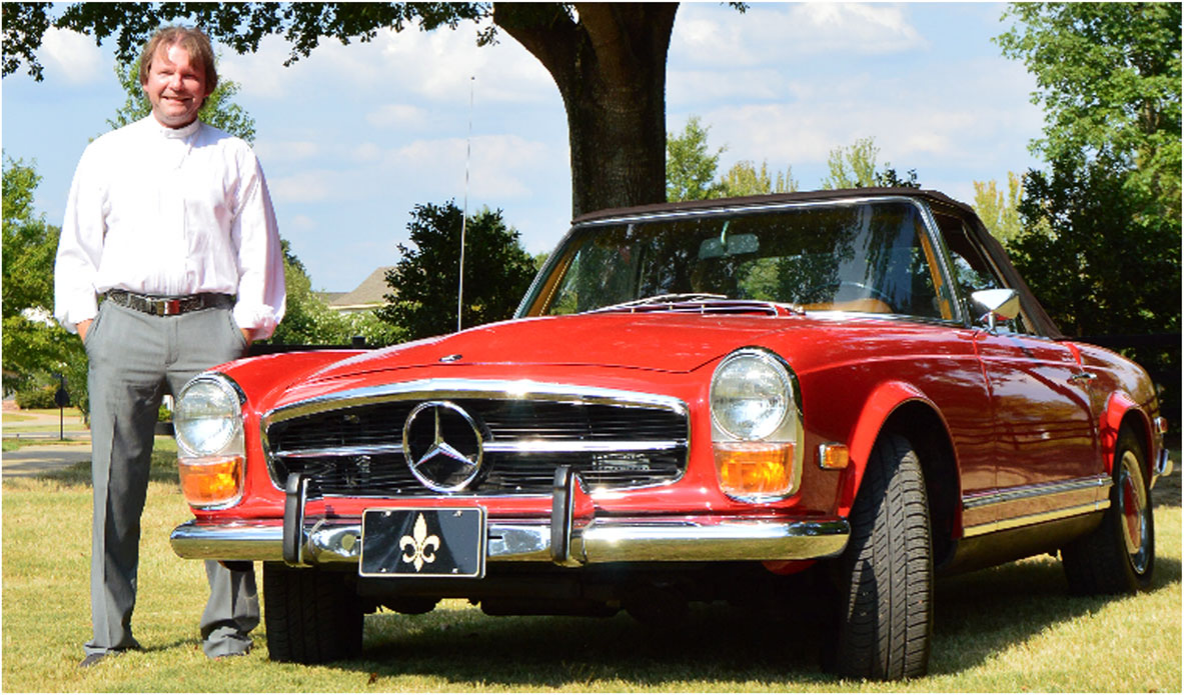
Prof. Christian Koch, deputy editor at *Journal of Medical Case Reports* since 2007

With the help of the editorial staff, *JMCR* has become a success, with increasing submissions from all over the world. Initially residing in Australia when *JMCR* was launched, editor Prof. Kidd has now relocated to Toronto, Canada. I myself was born and raised in Nuremberg, Germany, and worked in the USA last from 2006 to May 2017 before relocating back to Germany. Since returning, I have encountered there an attitude change in physicians, with now less than 50% of medical doctors completing a dissertation compared to 25 years ago. Many younger physicians, but also, increasingly, older physicians, no longer trust statistical truth finder studies with conclusions that simply do not match up with the medical realities in individual patient care. On the other hand, many contemporary “scientific” medical studies with many known and unknown variables confirm what was already known, or is common sense, similar to the fact that one should typically not cross a red traffic light because an accident might happen (Shanks JA. Probability in action: the red traffic light. *Journal of Statistical Education*. 2007, Volume 15, Issue 1. https://www.tandfonline.com/doi/full/10.1080/10691898.2007.11889457).

As stated by me in 2009 (https://casesnetwork.wordpress.com/2009/05/01/christian-koch-interview/), we are in an era of “evidence-based medicine.” However, we have finally begun to realize that much of the so-called evidence is derived from heterogeneous patient populations and is not necessarily applicable to our individual patients we see on a daily basis. Numerous studies including ENHANCE, ACCORD, ADVANCE, the VA Diabetes Trial, and SPRINT have shown us that heterogeneity of patients, equipment, infrastructure, investigators, and so on can limit the successful gathering of evidence on a large scale; this makes us think about personalized, individualized medicine and that is why publishing case reports is important [3–5]. Many years ago, Prof. Ioannidis has provided insight why most published research findings are false [6]. In part, reasons for that lie in the way funding networks are set up / arranged which often does not promote innovation and real discovery, although this is frequently pretended. In times with limited science funding, reassessing the grading of scholarly productivity and the way scientists and clinician-scientists should be evaluated is in order to preserve academic institutions [7, 8]. “Medical science politicians,” however, love to use trendy phrases like “patient explorer data warehouse,” “outcomes research,” and “big data analysis” without having learnt or, worse, despite knowing better, that the equation still remains “garbage in = garbage out.” Data entry depends on a well-trained and highly precise medical professional with a mindset that is able to think outside the box. All this starts with fully understanding the medical condition of ONE individual patient and I strongly doubt that many of these grant-seeking “medical science politicians” have ever attempted to simply do that: completely understand everything about ONE patient and record it. The truth of clinical success is always tested in an *N*=1 patient; our credibility and trust as physicians is on the line with the one patient we see on the other side of the room. It is then and there that our training, years of dedication and hard work, experience, and clinical insight are tested, each day and every day.

The editor of *JMCR*, Prof. Kidd, not only had that vision but acted on it and launched *JMCR* in 2007 with obvious ongoing success [9–12]. Many other publishers followed suit and launched spin-off journals to publish case reports separately (*American Journal of Case Reports, BMJ Case Reports*, and many others). Conducting retrospective studies with less than 100 patients early in my academic career, I felt frustration at having to list every single case in table form and then perform statistics on this data because journal reviewers requested such analyses be done before articles were acceptable for publication. Worse, I encountered basic science researchers who conducted statistical analyses on seven or eight rat study samples, published such studies in high-impact journals, and then really thought of themselves as great scientists.

After leaving Germany to obtain residency training at the outstanding and comprehensive Ohio State University Medical Center, USA, under the chairmanship of Prof. Ernest Mazzaferri, I had the good fortune to work with excellent medical colleagues who always tried to fully understand the ONE patient [13–17]. I then had the honour to join the prestigious research team around my mentor Prof. George Chrousos at the National Institutes of Health in Bethesda, Maryland, USA [18–23]. There, I learned how to conduct laboratory science studies and apply them to the individual patient [24–29]. I remember vividly the endorphin rush running through my arteries when we had a case study accepted in the *New England Journal of Medicine* after the editor Dr Robert Utiger had corrected the manuscript [30]. After returning to Germany in 2002 to take on a highly prestigious state government position and completing the habilitation procedure to become eligible for a full professorship, I continued teaching medical students, interns, residents, fellows, and working with faculty members valuing the full and uncompromising understanding of one individual patient [31, 32]. At that time, I had the pleasure to meet with a small group of outstanding clinical science endocrinologists who shared my view [33, 34], and we quickly discovered major “research studies,” published in prestigious medical journals, that should perhaps be classified as “*Mogelpackung*” (research making big claims nicely wrapped up in a neat package). In 2006, I then returned to the USA to take on a position as full professor and director of the Division of Endocrinology, Diabetes, and Metabolism at the University of Mississippi Medical Center (UMMC). The challenge and opportunity there was the “open range” and evolving infrastructure. I could, therefore, become active in a wide array of endocrinological aspects and research and successfully launched and concluded several studies, including case studies [35–40]. What fellow physicians and scientists need to understand is that textbook knowledge often cannot be applied to socioeconomically challenged communities with many patients presenting with advanced disease states that very few medical doctors have ever previously encountered in real life or in the medical literature. As much as I could, I tried to promote the vision of *JMCR* and assisted in recruiting several UMMC faculty members to become associate editors, namely Drs Garla, Lien, Pound, Yanes, and Vick.

Around the time of my arrival at UMMC, Prof. Tibor Fülöp (Fig. 2) also joined the Medical Center as a faculty member and we started collaborating on various projects [41–45]. Prof. Fülöp is an outstanding internist, nephrologist, educator, and researcher who has the ability to think outside the box. It is therefore with great pleasure that I introduce him as a new deputy editor for *JMCR*. Prof. Fülöp has risen through the academic ranks from assistant professor, to associate professor, to full professor with tenure at UMMC. He then returned to his native Hungary to complete his PhD degree in 2015. Afterward, he joined the Medical University of South Carolina in the USA as a full professor of medicine in the Department of Medicine. I wish him the best of success in his journey with *JMCR* and I am hopeful that he will enjoy the ride as much as I did, or more. 

In conclusion, I would like to remind all potential authors that *JMCR* does not only accept the zebras and black swans among patient encounters [46] but also follows the listed criteria (https://jmedicalcasereports.biomedcentral.com/about):

**Fig. 2:**
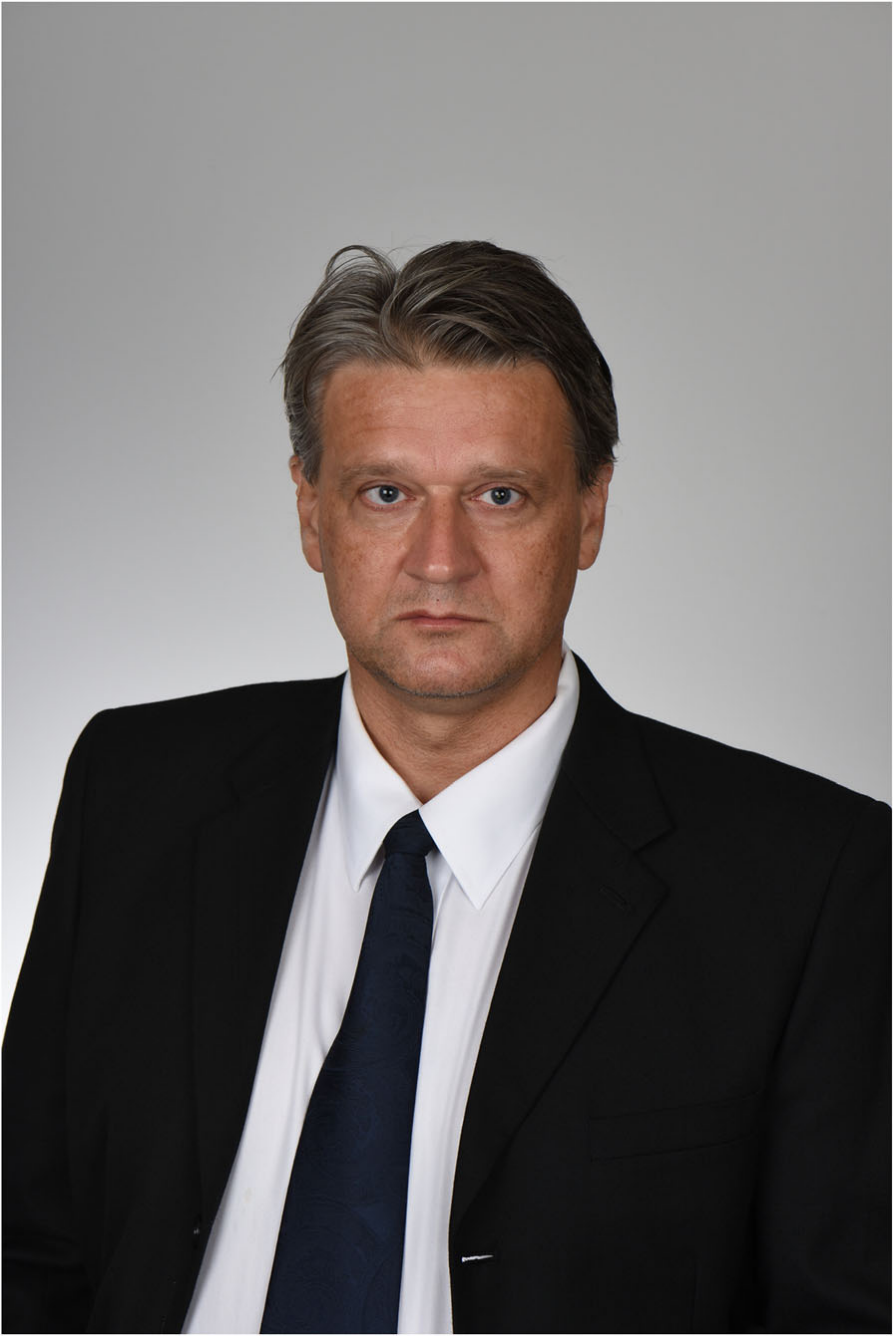
Prof. Tibor Fülöp, Medical University of South Carolina, USA

Case reports should show one of the following:

- Unreported or unusual side effects or adverse interactions involving medications.
- Unexpected or unusual presentations of a disease.
- New associations or variations in disease processes.
- Presentations, diagnoses, and/or management of new and emerging diseases.
- An unexpected association between diseases or symptoms.
- An unexpected event in the course of observing or treating a patient.
- Findings that shed new light on the possible pathogenesis of a disease or an adverse effect.

Suitable research articles include but are not limited to: *N* of 1 trials, meta-analyses of published case reports, research addressing the use of case reports and the prevalence or importance of case reporting in the medical literature, and retrospective studies that include case-specific information (age, sex, and ethnicity) for all patients.

So long and many thanks for the ride!

Christian Albert Koch.

## Authors’ information

Prof. Koch is affiliated with the Carl von Ossietzky University as well as the Technical University of Dresden, MK III (Chair, Prof. Stefan Bornstein), Germany. He also is adjunct professor at the University of Louisville, KY, USA, and works at Medicover GmbH Germany. Before June 2017, Koch had served as a professor of medicine (with tenure) at the University of Mississippi Medical Center in Jackson, MS, USA, and staff physician at the G.V. (Sonny) Montgomery VA Medical Center. He is a “*summa cum laude*” graduate of the Friedrich-Alexander University in Erlangen-Nuremberg, Germany and trained in neurosurgery, neurology, and internal medicine in Germany. He then completed a residency in Internal Medicine at the Ohio State University in Columbus, OH, USA, under the chairmanship of Prof. EL Mazzaferri, and clinical and research training in endocrinology at the National Institutes of Health in Bethesda, MD, USA, under the guidance of Prof. George P. Chrousos and many collaborating colleagues. Prof. Koch’s research focus has been the pathogenesis of endocrine tumors and endocrine hypertension. He remains interested in translational and multidisciplinary research with questions arising from patient encounters, trying to explain clinical observations by looking at bench results to further improve patient care. In May 2017, the American College of Endocrinology awarded Prof. Koch the prestigious title of Master of the American College of Endocrinology (MACE) (https://www.umc.edu/news/Miscellaneous/2017/May/Endocrinologist-to-receive-prized-MACE--grad-students--fellows-earn-biology-honors.html).
